# Composite Inflammatory Score for Predicting Progression‐Free Survival in Metastatic Clear Cell Renal Cell Carcinoma Treated With First‐Line Toripalimab Plus Axitinib

**DOI:** 10.1002/kjm2.70307

**Published:** 2026-10-22

**Authors:** Yun‐Feng Wu, Jing‐Suo Wang, Chuan‐Ao Zhang, Fan Zhang, Zhi‐Yu Zhang

**Affiliations:** ^1^ Department of Urology The First People's Hospital of Jintan Changzhou Jiangsu China; ^2^ Department of Urology The First Affiliated Hospital of Soochow University Suzhou Jiangsu China; ^3^ Department of Nephrology First Affiliated Hospital of Soochow University Suzhou Jiangsu China

**Keywords:** immune checkpoint inhibitors, inflammation, prognosis, progression‐free survival, renal cell carcinoma

## Abstract

Routine inflammatory and nutritional markers may help stratify progression risk in metastatic clear cell renal cell carcinoma during immunotherapy‐based treatment. This single‐center retrospective study included 237 patients who received first‐line toripalimab plus axitinib therapy. Ten indices were natural‐log transformed and standardized, and domain‐based selection yielded a compact composite inflammatory score (COMP‐IS), which was internally validated using 1000 full‐pipeline bootstrap resamples. The primary endpoint was progression‐free survival (PFS). During a median follow‐up of 19.84 months for PFS, 124 events occurred. COMP‐IS comprised the neutrophil‐to‐lymphocyte ratio, C‐reactive protein‐to‐albumin ratio (CAR), and prognostic nutritional index. After adjusting for the International Metastatic Renal Cell Carcinoma Database Consortium (IMDC) score, each standard deviation increase in COMP‐IS was associated with shorter PFS (hazard ratio, 3.48; 95% confidence interval, 2.74–4.42). The apparent concordance index (C‐index) for COMP‐IS was 0.767, with a bootstrap‐corrected C‐index of 0.755, compared with corrected C‐indices of 0.694 for CAR and 0.565 for the IMDC score. Adding IMDC to COMP‐IS resulted in a corrected C‐index of 0.756. In an exploratory post hoc comparison, the published Meet‐URO risk groups had an apparent C‐index of 0.593. COMP‐IS demonstrated prognostic discrimination for PFS in this cohort; however, only internal validation was performed. Its clinical utility therefore remains uncertain, and independent external validation and prospective evaluation are needed before routine clinical use.

## 1 Introduction

Immune checkpoint inhibitor (ICI)–based combinations have redefined first‐line therapy for metastatic clear cell renal cell carcinoma (mccRCC). In the RENOTORCH trial, toripalimab plus axitinib improved progression‐free survival (PFS) and objective response versus sunitinib. These findings support using a combination of ICI with a vascular endothelial growth factor receptor tyrosine kinase inhibitor (TKI) in Chinese patients [1]. Extended follow‐up has also confirmed durable benefits with pembrolizumab plus axitinib, nivolumab plus cabozantinib, and lenvatinib plus pembrolizumab [2, 3, 4]. However, treatment outcomes remain heterogeneous, with patients receiving the same combination experiencing durable disease control, early progression, or treatment‐limiting clinical deterioration. A readily calculable prognostic measure could improve baseline risk assessment and guide follow‐up intensity without requiring additional tissue sampling or specialized molecular platforms.

The International Metastatic Renal Cell Carcinoma Database Consortium (IMDC) model is a well‐established clinical prognostic framework [5]. Its six components capture performance status, disease tempo, anemia, hypercalcemia, neutrophilia, and thrombocytosis; however, the model was developed during the era of vascular endothelial growth factor–targeted therapy and only indirectly reflects systemic inflammatory and nutritional status. The Meet‐URO score extends this framework by incorporating pretreatment neutrophil‐to‐lymphocyte ratio (NLR) and the presence of bone metastases [6]. It has been evaluated in cohorts receiving first‐line ICI‐based combination therapy, including a post hoc analysis of the prospective ProPAXI study of pembrolizumab plus axitinib [7, 8]. Contemporary reviews emphasize that no routinely used biomarker currently guides the selection of first‐line ICI‐based combinations [9]. Host physiological and nutritional status may provide prognostic information not fully captured by categorical clinical risk models, as supported by body composition studies of patients receiving immune checkpoint blockade [10]. Blood cell counts and C‐reactive protein (CRP), albumin, and fibrinogen are therefore attractive candidate markers because they are routinely available and reproducible in clinical practice.

A key challenge is the substantial overlap among candidate indices. NLR, derived neutrophil‐to‐lymphocyte ratio (dNLR), systemic immune‐inflammation index (SII), and systemic inflammation response index (SIRI) share neutrophil‐ and lymphocyte‐based components. In addition, platelet‐to‐lymphocyte ratio (PLR) and SII incorporate platelet counts, while lymphocyte‐to‐monocyte ratio (LMR) and SIRI incorporate monocyte counts. The C‐reactive protein‐to‐albumin ratio (CAR) and fibrinogen‐to‐albumin ratio (FAR) combine acute‐phase reactants with albumin, whereas the prognostic nutritional index (PNI) and advanced lung cancer inflammation index (ALI) reflect nutritional and systemic inflammatory status. Meta‐analyses and contemporary renal cell carcinoma (RCC) cohorts have demonstrated prognostic associations for these and related indices during systemic or ICI‐based treatments [11, 12, 13, 14, 15]. NLR and CRP‐based measures have also shown prognostic value in first‐line ICI–TKI cohorts, while modified Glasgow prognostic scores and early CRP kinetics further support the prognostic relevance of systemic inflammation during treatment [16, 17, 18, 19, 20]. However, differences in study populations, variable transformations, and data‐derived cutoffs limit comparisons across studies. Rigorous comparison requires consistent continuous‐variable processing, direct benchmarking against the best‐performing individual marker, and internal validation of the entire data‐dependent modeling pipeline rather than the final model alone.

In this study, we compared 10 routine inflammatory and nutritional indices under a common continuous‐variable framework in patients receiving first‐line toripalimab plus axitinib and constructed a compact composite inflammatory score (COMP‐IS) from complementary biological domains. All eligible patients contributed to model development to maximize information from the available events. Overfitting and model selection instability were evaluated using a full‐pipeline bootstrap internal validation. We assessed associations with PFS, discrimination relative to individual indices, IMDC, and Meet‐URO, time‐dependent accuracy, calibration, overall prediction error, decision curves, stratification using a fixed risk threshold, secondary response and safety outcomes, and exploratory subgroup consistency. Because the cohort lacked an alternative treatment arm, this study addressed prognosis during therapy rather than differential treatment benefits.

## 2 Methods

### 2.1 Study Design, Setting, and Reporting

This retrospective cohort study was conducted at our hospital. Consecutive eligible patients initiating first‐line toripalimab plus axitinib therapy from June 1, 2024, through June 30, 2025, were included, with follow‐up closed on June 30, 2026. All 237 analyzed patients formed a single model development cohort, and no calendar or random development–validation split was used. Internal validation was performed using bootstrap resampling as described below. Reporting follows the Transparent Reporting of a multivariable prediction model for Individual Prognosis or Diagnosis, extended to artificial intelligence (TRIPOD + AI) statement, which applies to both regression and machine learning prediction models [21]. The Ethics Committee of our institution approved the study (No. 682(2026)). The requirement for written informed consent was waived because the study was retrospective and used de‐identified data.

### 2.2 Participants

Eligible patients were aged 18 years or older; had histologically confirmed clear cell RCC that was unresectable or metastatic; received toripalimab plus axitinib as first‐line systemic therapy; had Eastern Cooperative Oncology Group (ECOG) performance status (PS) 0–2; had at least one measurable lesion; had complete blood count, CRP, albumin, fibrinogen, and other required biochemical measurements within 7 days before treatment; and underwent at least one post‐treatment efficacy assessment. Exclusion criteria were nonclear cell histology or mixed histology with more than 50% sarcomatoid differentiation, previous systemic anticancer therapy except adjuvant or neoadjuvant treatment followed by relapse more than 12 months later, active infection or autoimmune disease, systemic glucocorticoid exposure exceeding 10 mg/day prednisone equivalent within 1 month before treatment, another malignancy within 5 years, a hematologic disorder, absence of variables required for score or endpoint calculation, or loss to follow‐up before efficacy assessment. Aggregate screening identified 263 patients, 26 of whom were excluded. No missing values remained in the final analysis dataset and no imputation was performed.

### 2.3 Treatment, Assessments, and Clinical Variables

Toripalimab was administered intravenously at 240 mg every 3 weeks. Axitinib was administered orally at 5 mg twice daily and adjusted according to toxicity in routine practice. Clinical review and laboratory testing were generally performed every 3 weeks, and contrast‐enhanced chest, abdominal, and pelvic computed tomography was generally performed every 3 months or earlier, when clinically indicated. Responses were classified according to the Response Evaluation Criteria in Solid Tumors (RECIST), version 1.1 [22]. Adverse events were graded using the Common Terminology Criteria for Adverse Events (CTCAE), version 5.0, with the highest grade recorded during treatment. IMDC assigned one point each for Karnofsky performance status below 80%, diagnosis‐to‐treatment interval below 1 year, hemoglobin below the local lower limit of normal, corrected calcium above the local upper limit, neutrophils above the upper limit, and platelets above the upper limit; scores of 0, 1–2, and ≥ 3 defined favorable, intermediate, and poor risk, respectively [5].

Meet‐URO was calculated using published weights: 0, 3, or 6 points for favorable, intermediate, or poor IMDC risk, respectively; 2 points for NLR ≥ 3.2; and 1 point for bone metastases. The original five risk groups corresponded to total scores of 0–1, 2–3, 4–5, 6–8, and 9, respectively [6]. This established score was evaluated as an additional comparator and was not included in the domain‐based selection of the candidate indices.

### 2.4 Inflammatory and Nutritional Indices

Pretreatment laboratory measurements were obtained within 7 days before treatment initiation. CRP was measured in the serum collected in serum separator tubes using a particle‐enhanced immunoturbidimetric assay on a Beckman Coulter AU5800 automated chemistry analyzer (Beckman Coulter Inc., Brea, CA, USA). Fibrinogen was measured in platelet‐poor citrated plasma (3.2% trisodium citrate; 9:1 blood‐to‐anticoagulant ratio) using a Clauss clotting assay on a Sysmex CS‐5100 automated coagulation analyzer (Sysmex Corporation, Kobe, Japan). Assays were calibrated using manufacturer‐matched calibrators at manufacturer‐recommended intervals and whenever the reagent lot was changed. Daily internal quality control used two‐level commercial control materials and the Westgard multirule criteria. The laboratory participated in the external quality assessment program of the National Centre for Clinical Laboratories (National Health Commission of China) with satisfactory results. Both the analytical platforms and assay methods remained unchanged throughout the study period; therefore, no between‐platform comparability assessments were required.

The indices were calculated using absolute neutrophil (*N*), lymphocyte (*L*), monocyte (*M*), platelet (*P*), and white blood cell (*W*) counts (all in ×10^9^/L), together with albumin (*A*, g/L), CRP (*C*, mg/L), fibrinogen (*F*, g/L), and body mass index (*B*, kg/m^2^). The 10 candidate indices were NLR = *N*/L; PLR = *P*/L; LMR = L/M; SII = P × *N*/L; SIRI = M × *N*/L; dNLR = *N*/(W − *N*); PNI = A + 5 L; CAR = C/A; FAR = F/A; and ALI = B × A/NLR. Each candidate was retained as a continuous variable. To ensure identical processing, every index was natural‐log transformed and standardized to a mean of 0 and a sample standard deviation (SD) of 1 in the full development cohort. No marker‐specific transformation or outcome‐derived dichotomization was used.

### 2.5 Candidate Comparison, Selection, and Composite Inflammatory Score Construction

Each standardized candidate was fitted separately in a Cox proportional hazards model for PFS [23]. Candidates were grouped according to hematologic inflammation (NLR, PLR, LMR, SII, SIRI, and dNLR), acute phase/nutritional balance (CAR and FAR), and integrated nutritional reserve (PNI and ALI). The lowest Akaike information criterion (AIC) was identified within each domain. When another candidate was within two AIC units, the index requiring fewer distinct raw laboratory or anthropometric components was selected; the remaining ties were resolved using a lower AIC. NLR, CAR, and PNI were selected using this procedure. Their full‐cohort standardization was: z_NLR_ = [ln(NLR) − 1.001470843]/0.446175333; *z*_CAR_ = [ln(CAR) + 2.081861569]/0.980723074; *z*_PNI_ = [ln(PNI) − 3.862509677]/0.099894837. The three standardized inputs were fitted together in a Cox model, yielding *R* = 0.669085458*z*_NLR_ + 0.658801143*z*_CAR_ − 0.454941002*z*_PNI_. The score was then standardized as COMP‐IS = (*R* − 1.885e − 15)/1.264694259. The centering term is a numerical round‐off around zero. Thus, one COMP‐IS unit corresponds to one full‐cohort SD of the composite linear predictor. For descriptive risk stratification, COMP‐IS ≥ 1.0 defined COMP‐IS‐High; this rule represents one SD above the full‐cohort mean and was not optimized against survival outcomes.

### 2.6 Endpoints

The primary endpoint was PFS, measured from the first treatment administration to radiographic progression using RECIST version 1.1, or death from any cause, whichever occurred first. Patients without events were censored at the last disease assessment confirming the absence of disease progression. Secondary endpoints were overall survival (OS), objective response rate (ORR; complete plus partial response), disease control rate (DCR; complete response, partial response, or stable disease), grade ≥ 3 adverse events, immune‐related adverse events, axitinib dose reduction, and treatment discontinuation attributable to adverse events. OS was measured from the first treatment administration to death from any cause. Patients who were alive were censored at the last date they were known to be alive.

### 2.7 Statistical Analysis and Internal Validation

Continuous variables are summarized as median (interquartile range) and categorical variables as *n* (%). Cox models used Efron handling of tied event times and reported hazard ratios (HRs) with 95% confidence intervals (CIs) [23]. All eligible patients during the study period were included, and model complexity was constrained, given the available number of events, rather than justified by a simple events‐per‐variable rule [24]. Proportional hazards assumptions were evaluated using Schoenfeld residuals and their association with the log‐transformed event time [25]. Kaplan–Meier estimates and log‐rank tests were used for the fixed‐threshold analysis [26]. The OS probabilities at 12 and 24 months were estimated for the full cohort using the Kaplan–Meier method, with pointwise 95% CIs calculated using Greenwood's variance and a log–log transformation. The numbers of patients remaining at risk at these time points were also reported. The median follow‐up period was estimated separately for PFS and OS using the reverse Kaplan–Meier method. Discrimination was quantified with Harrell's concordance index (C‐index) [27]. Patient‐level bootstrap resampling was used for C‐index confidence intervals and paired comparisons [28]. Cumulative/dynamic time‐dependent areas under the curve (AUCs) were estimated for clinically relevant horizons [29]. Calibration was evaluated at 6, 9, and 12 months using grouped observed Kaplan–Meier probabilities, calibration slope, and inverse probability of censoring‐weighted (IPCW) Brier scores [30]. Exploratory decision curve analysis (DCA) assessed the net benefit of model‐predicted progression or death by 12 months over threshold probabilities of 0.10–0.60 [31]. The analysis used model‐derived absolute event risks rather than the fixed COMP‐IS ≥ 1.0 classification. Specific COMP‐IS‐guided interventions or action‐specific thresholds were not evaluated.

For clinical anchoring, the combined absolute‐risk model included continuous COMP‐IS and continuous IMDC scores, irrespective of the coefficient *p*‐value for IMDC, in alignment with the established clinical prognostic framework rather than significance‐driven predictor retention. Internal validation used 1000 nonparametric full‐pipeline bootstrap resamples. In every resample, all 10 indices were re‐transformed and re‐standardized using bootstrap‐specific constants; all 10 candidate Cox models were refitted, domain‐based AIC selection was repeated, the three‐variable COMP‐IS was reconstructed and standardized, and the COMP‐IS and IMDC+COMP‐IS models were refitted. Differences in performance between the bootstrap sample and the original cohort under each bootstrap‐derived model yielded optimism estimates, which were averaged and subtracted from the apparent performance. The same full‐pipeline principle was applied to the 6‐, 9‐, and 12‐month AUCs. The selection frequencies across bootstrap resamples quantified the instability of the exact marker composition. The calibration slope reported for the final model was internally corrected by applying bootstrap‐fitted models to the original cohort. Tests were two‐sided. Analyses were performed using Python 3.13.5, NumPy 2.3.5, Pandas 2.2.3, SciPy 1.17.0, Statsmodels 0.14.6, Matplotlib 3.10.8, and Pillow 12.2.0. The random seed was 20,260,807.

In an additional post hoc analysis, the published Meet‐URO risk groups were used as a fixed ordinal risk ranking for PFS without reestimating the category coefficients or cutoffs. A total score of 0–9 and three IMDC risk categories were examined in the sensitivity analyses. C‐indices; 6‐, 9‐, and 12‐month AUCs; and paired differences in the apparent C‐index were evaluated using 1000 patient‐level bootstrap resamples with percentile 95% CIs (seed 20,260,807). All scores were fixed in these additional resamples; the resulting intervals were conditional on the final scores and did not incorporate the COMP‐IS model selection uncertainty or provide an optimism correction. These exploratory comparisons were interpreted separately from the full‐pipeline validation described above. For PFS, no patient was censored before 12 months; therefore, the time‐dependent AUCs at these horizons were reduced to case–control comparisons without nontrivial censoring weights. Additional analyses were performed using Python 3.12.14, NumPy 2.3.5, and Pandas 2.2.3.

## 3 Results

### 3.1 Cohort Characteristics

Of the 263 screened patients, 26 were excluded and 237 were analyzed (Figure 1). The cohort included 124 PFS events, and the reverse Kaplan–Meier median follow‐up period for PFS was 19.84 months. The median PFS in the full cohort was 16.69 months. The baseline characteristics according to the fixed COMP‐IS category are summarized in Table 1. The high‐risk group included 35 patients (14.8%).

**FIGURE 1:**
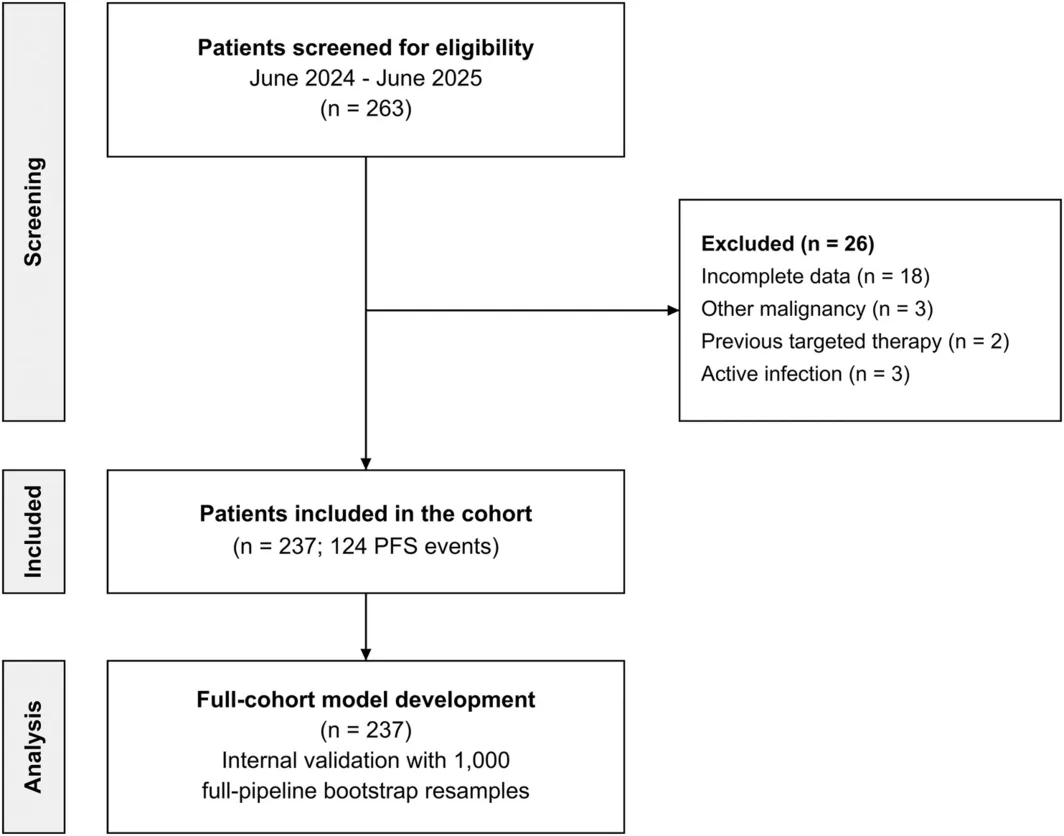
Patient selection and full‐cohort model‐development strategy. The diagram summarizes patient screening, reasons for exclusion, and inclusion in the analysis cohort. All 237 included patients contributed to model development; internal validation used 1000 full‐pipeline bootstrap resamples. Screening exclusions are aggregate counts from the institutional screening record. PFS, progression‐free survival.

**TABLE 1:** Baseline characteristics according to the fixed COMP‐IS category.

Characteristics	Level	Overall (*N* = 237)	COMP‐IS‐Low (*n* = 202)	COMP‐IS‐High (*n* = 35)	*p*
Age, years		60 (54–67)	60 (54–67)	57 (54–62)	0.188
Sex	Male	176 (74.3%)	151 (74.8%)	25 (71.4%)	0.679
	Female	61 (25.7%)	51 (25.2%)	10 (28.6%)	
BMI, kg/m^2^		23.4 (21.2–25.9)	23.4 (21.2–25.8)	23.1 (20.9–26.1)	0.685
ECOG performance status	0	55 (23.2%)	41 (20.3%)	14 (40.0%)	0.038
	1	145 (61.2%)	128 (63.4%)	17 (48.6%)	
	2	37 (15.6%)	33 (16.3%)	4 (11.4%)	
Prior nephrectomy	Yes	161 (67.9%)	134 (66.3%)	27 (77.1%)	0.243
	No	76 (32.1%)	68 (33.7%)	8 (22.9%)	
Metastatic organs, *n*		2 (1–2)	2 (1–2)	2 (1–2)	0.531
IMDC risk group	Favorable	60 (25.3%)	51 (25.2%)	9 (25.7%)	0.769
	Intermediate	174 (73.4%)	148 (73.3%)	26 (74.3%)	
	Poor	3 (1.3%)	3 (1.5%)	0 (0.0%)	
Lung metastasis	Yes	146 (61.6%)	130 (64.4%)	16 (45.7%)	0.040
	No	91 (38.4%)	72 (35.6%)	19 (54.3%)	
Bone metastasis	Yes	65 (27.4%)	54 (26.7%)	11 (31.4%)	0.545
	No	172 (72.6%)	148 (73.3%)	24 (68.6%)	
Liver metastasis	Yes	46 (19.4%)	38 (18.8%)	8 (22.9%)	0.643
	No	191 (80.6%)	164 (81.2%)	27 (77.1%)	
NLR		2.75 (2.01–3.86)	2.55 (1.86–3.37)	4.30 (3.79–5.16)	< 0.001
CAR		0.124 (0.063–0.240)	0.106 (0.057–0.189)	0.411 (0.222–0.601)	< 0.001
PNI		47.8 (45.0–50.6)	48.3 (45.9–50.9)	42.6 (39.9–46.1)	< 0.001

*Note:* Continuous variables are presented as medians (interquartile ranges) and categorical variables as *n* (%). *p*‐Values are descriptive. The cohort includes 124 PFS events, and reverse Kaplan–Meier median follow‐up for PFS is 19.84 months.

Abbreviations: BMI, body mass index; CAR, C‐reactive protein‐to‐albumin ratio; COMP‐IS, composite inflammatory score; ECOG, Eastern Cooperative Oncology Group; IMDC, International Metastatic Renal Cell Carcinoma Database Consortium; NLR, neutrophil‐to‐lymphocyte ratio; PFS, progression‐free survival; PNI, prognostic nutritional index.

### 3.2 Comparison of 10 Candidate Indices and Model Selection

All 10 continuously modeled indices were associated with PFS in the univariate Cox analyses (Table 2). The strongest individual discrimination was observed for CAR, whereas leukocyte‐derived ratios showed modest performance. NLR had the lowest AIC within the hematologic domain. CAR was preferred over FAR in the acute phase domain. In the nutritional domain, ALI had the lowest AIC, but PNI was within two AIC units and was retained by the complexity rule. Full‐pipeline bootstrap selection supported the NLR as a stable hematologic representative (93.1% of resamples), whereas the acute‐phase and nutritional representatives were less stable: CAR was selected in 62.6%, FAR in 37.4%, PNI in 47.9%, and ALI in 52.1%. The selected NLR, CAR, and PNI inputs exhibited low collinearity (all variance inflation factors < 1.3).

**TABLE 2:** Common‐framework comparison of 10 candidate inflammatory and nutritional indices.

Index	Domain	HR (95% CI)	*p*	Apparent C‐index (95% CI)	Corrected C‐index	6 m AUC	9 m AUC	12 m AUC	Bootstrap selection frequency	COMP‐IS
NLR	Inflammation	1.97 (1.60–2.42)	< 0.001	0.670 (0.618–0.722)	0.669	0.694	0.716	0.723	93.1%	Selected
PLR	Inflammation	1.43 (1.19–1.72)	< 0.001	0.601 (0.548–0.655)	0.600	0.618	0.631	0.636	0.0%	
LMR	Inflammation	0.67 (0.56–0.81)	< 0.001	0.610 (0.565–0.661)	0.609	0.633	0.634	0.644	0.3%	
SII	Inflammation	1.85 (1.50–2.29)	< 0.001	0.655 (0.603–0.704)	0.654	0.673	0.697	0.704	4.4%	
SIRI	Inflammation	1.80 (1.47–2.20)	< 0.001	0.647 (0.594–0.697)	0.645	0.667	0.682	0.690	2.2%	
dNLR	Inflammation	1.84 (1.51–2.24)	< 0.001	0.661 (0.605–0.713)	0.660	0.685	0.709	0.714	0.0%	
PNI	Nutritional	0.48 (0.40–0.58)	< 0.001	0.682 (0.637–0.727)	0.681	0.742	0.743	0.743	47.9%	Selected
CAR	Mixed	1.98 (1.64–2.41)	< 0.001	0.694 (0.648–0.741)	0.694	0.735	0.747	0.747	62.6%	Selected
FAR	Mixed	1.96 (1.59–2.41)	< 0.001	0.683 (0.635–0.726)	0.683	0.757	0.755	0.745	37.4%	
ALI	Nutritional	0.46 (0.37–0.56)	< 0.001	0.684 (0.634–0.730)	0.682	0.715	0.733	0.740	52.1%	

*Note:* All indices are natural‐log‐transformed and z‐standardized using full‐cohort means and sample SDs. Bootstrap‐corrected C‐index repeats candidate processing and model fitting. For CAR, the apparent C‐index CI is reported from the principal benchmark analysis (Table 3). AUC columns show full‐cohort estimates at 6, 9, and 12 months; corrected AUCs for the principal benchmark models are reported in Table 3. Selection frequency is based on 1000 full‐pipeline bootstrap resamples. Domain selection uses AIC; when ΔAIC ≤ 2, the formula requiring fewer distinct raw components is selected. VIFs for NLR, CAR, and PNI are 1.10, 1.18, and 1.28, respectively.

Abbreviations: AIC, Akaike information criterion; ALI, advanced lung cancer inflammation index; AUC, area under the curve; C‐index, concordance index; CAR, C‐reactive protein‐to‐albumin ratio; CI, confidence interval; COMP‐IS, composite inflammatory score; dNLR, derived neutrophil‐to‐lymphocyte ratio; FAR, fibrinogen‐to‐albumin ratio; HR, hazard ratio; LMR, lymphocyte‐to‐monocyte ratio; NLR, neutrophil‐to‐lymphocyte ratio; PLR, platelet‐to‐lymphocyte ratio; PNI, prognostic nutritional index; SD, standard deviation; SII, systemic immune‐inflammation index; SIRI, systemic inflammation response index; VIF, variance inflation factor.

### 3.3 COMP‐IS Association and Internally Validated Discrimination

In the model containing COMP‐IS and IMDC score, each 1‐SD higher COMP‐IS was associated with shorter PFS (HR, 3.48; 95% CI, 2.74–4.42; *p* < 0.001). The adjusted IMDC estimate was HR 1.20 (95% CI, 0.96–1.50; *p* = 0.109; Table 3, Panel B). No proportional hazards violations were detected for the COMP‐IS or IMDC. The sensitivity model adjusted for age, prior nephrectomy, and metastatic organ count yielded similar COMP‐IS associations. The apparent C‐index for the COMP‐IS was 0.767 (95% CI, 0.728–0.803); after the 1000‐resample full‐pipeline optimism correction, it was 0.755. The corrected C‐index was 0.694 for the CAR and 0.565 for the IMDC. The apparent COMP‐IS‐versus‐CAR C‐index difference was 0.073 (95% CI, 0.033–0.113; *p* = 0.002), with a bootstrap‐corrected point difference of 0.062. The addition of IMDC to COMP‐IS changed the corrected C‐index by only 0.001 (Table 3, Panel C). The bootstrap‐corrected 6‐, 9‐, and 12‐month AUCs for COMP‐IS were 0.812, 0.833, and 0.836, respectively (Figure 2).

**TABLE 3:** COMP‐IS construction, multivariable association, internally validated model performance, fixed‐threshold stratification, and response outcomes.

Panel A. COMP‐IS construction and bootstrap selection stability			
Indicator	β in raw composite	Direction	Bootstrap selection frequency
NLR	0.669085	Adverse (higher value = higher risk)	93.1%
CAR	0.658801	Adverse (higher value = higher risk)	62.6%
PNI	−0.454941	Protective (higher value = lower risk)	47.9%

Panel B. Multivariable Cox model			
Variable	HR (95% CI)	*p*	PH‐test *p*
COMP‐IS	3.48 (2.74–4.42)	< 0.001	0.822
IMDC score	1.20 (0.96–1.50)	0.109	0.606

Panel C1. Model performance					
Model	Apparent C‐index (95% CI)	Corrected C‐index	Corrected 6 m AUC	Corrected 9 m AUC	Corrected 12 m AUC
CAR	0.694 (0.648–0.741)	0.694	0.735	0.747	0.748
IMDC	0.565 (0.516–0.616)	0.565	0.577	0.565	0.567
COMP‐IS	0.767 (0.728–0.803)	0.755	0.812	0.833	0.836
IMDC + COMP‐IS	0.770 (0.731–0.807)	0.756	0.811	0.831	0.833

Panel C2. Paired model comparisons			
Comparison	Apparent Δ*C*‐index (95% CI)	*p*	Corrected Δ*C*‐index
COMP‐IS vs. CAR	0.073 (0.033–0.113)	0.002	0.062
COMP‐IS vs. IMDC	0.202 (0.146–0.263)	< 0.001	0.191
IMDC + COMP‐IS vs. CAR	0.076 (0.034–0.119)	< 0.001	0.063
IMDC + COMP‐IS vs. COMP‐IS	0.003 (−0.004–0.010)	0.442	0.001
IMDC + COMP‐IS vs. IMDC	0.205 (0.156–0.258)	< 0.001	0.192

Panel D. Fixed‐threshold PFS stratification							
Group	N	Events	Median PFS, months (95% CI)	6 m PFS	9 m PFS	12 m PFS	Log‐rank *p*
COMP‐IS‐Low	202	90	22.42 (17.15–NR)	81.2%	74.8%	67.3%	< 0.001
COMP‐IS‐High	35	34	4.21 (2.61–6.35)	37.1%	14.3%	5.7%	

Panel E. Clinical response associations				
Endpoint	COMP‐IS‐Low	COMP‐IS‐High	OR high vs low (95% CI)	Fisher *p*
ORR	116/202 (57.4%)	10/35 (28.6%)	0.30 (0.14–0.65)	0.002
DCR	178/202 (88.1%)	21/35 (60.0%)	0.20 (0.09–0.45)	< 0.001

*Note:* *z*_NLR_ = [ln(NLR) – 1.001470843]/0.446175333; *z*_CAR_ = [ln(CAR) + 2.081861569]/0.980723074; *z*_PNI_ = [ln(PNI) – 3.862509677]/0.099894837. *R* = 0.669085458*z*_NLR_ + 0.658801143*z*_CAR_ – 0.454941002*z*_PNI_; COMP‐IS = (*R* – 1.885e‐15)/1.264694259. The centering term is numerical round‐off around zero. PNI was selected in the full cohort by the ΔAIC ≤ 2 complexity rule; ALI was selected in 52.1% of bootstrap resamples versus 47.9% for PNI, indicating instability within the nutritional‐reserve domain. The absolute‐risk model is LP = 1.247633749 × COMP‐IS + 0.183993889 × IMDC, with PFS(*t*|*x*) = *S*_0_(*t*)^exp(LP). Breslow baseline survival: *S*_0_ (6) = 0.855099990, *S*_0_ (9) = 0.775321485, *S*_0_ (12) = 0.690891590. The IMDC was retained in this absolute‐risk model as the established clinical prognostic anchor, irrespective of its coefficient *p*‐value; its inclusion was not based on significance‐driven variable selection. The internally corrected calibration slope was 0.928, and the apparent IPCW Brier scores were 0.136, 0.144, and 0.152 at 6, 9, and 12 months.

Abbreviations: AIC, Akaike information criterion; ALI, advanced lung cancer inflammation index; AUC, area under the curve; C‐index, concordance index; CAR, C‐reactive protein‐to‐albumin ratio; CI, confidence interval; COMP‐IS, composite inflammatory score; DCR, disease control rate; HR, hazard ratio; IMDC, International Metastatic Renal Cell Carcinoma Database Consortium; IPCW, inverse probability of censoring weighting; LP, linear predictor; NLR, neutrophil‐to‐lymphocyte ratio; NR, not reached; OR, odds ratio; ORR, objective response rate; PFS, progression‐free survival; PH, proportional hazards; PNI, prognostic nutritional index; SD, standard deviation.

**FIGURE 2:**
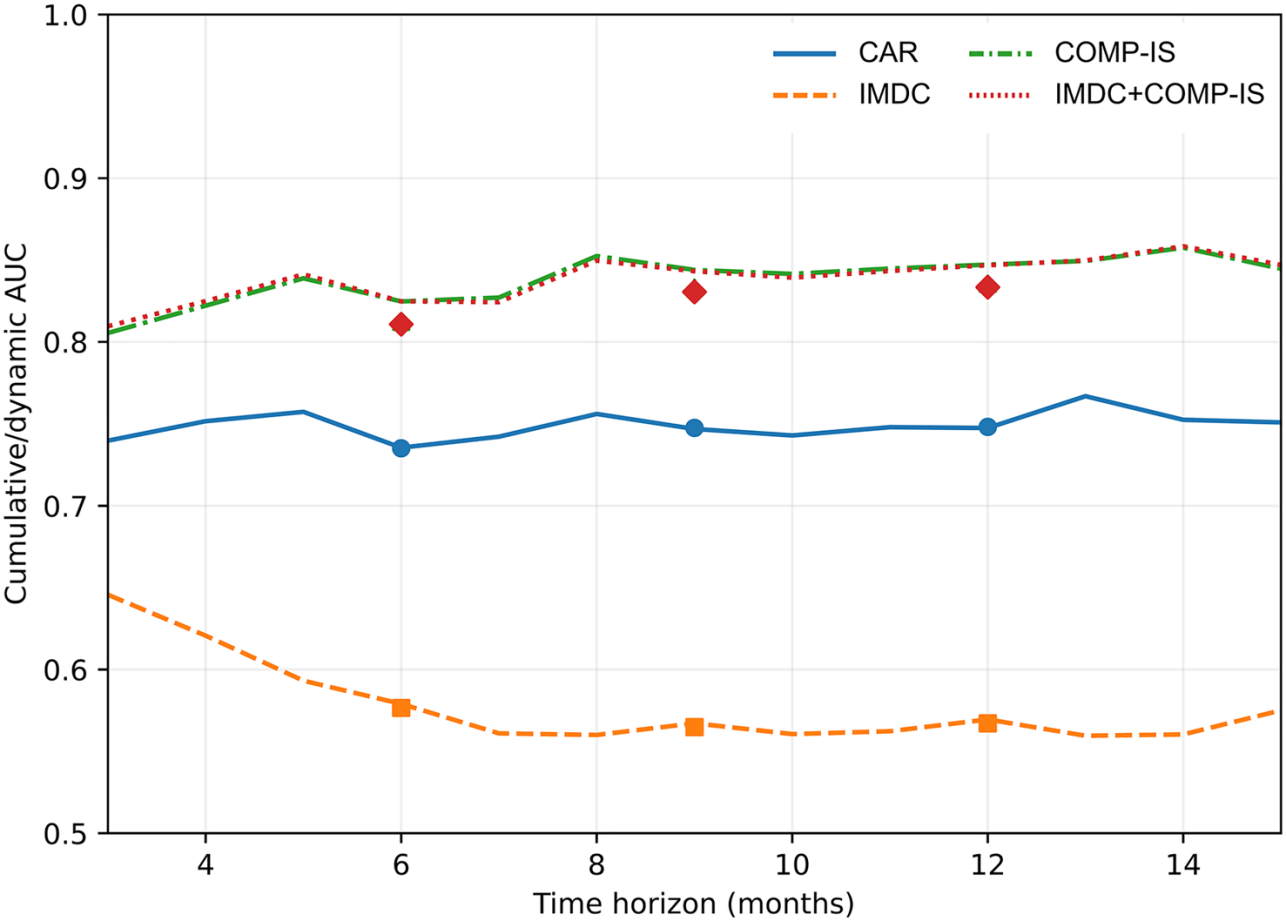
Time‐dependent discrimination from 3 to 15 months in the full cohort. Lines show the apparent cumulative/dynamic AUC trajectories for CAR, IMDC, COMP‐IS, and IMDC + COMP‐IS; model‐specific markers at 6, 9, and 12 months show the corresponding full‐pipeline bootstrap‐corrected AUCs. Models are color‐coded and additionally distinguished by line style and marker shape. AUC, area under the curve; CAR, C‐reactive protein‐to‐albumin ratio; COMP‐IS, composite inflammatory score; IMDC, International Metastatic Renal Cell Carcinoma Database Consortium.

In the post hoc comparison, Meet‐URO scores were available for all 237 patients; the 5 risk groups contained 39, 103, 72, 22, and one patient, respectively. The published risk groups had a C‐index of 0.593 (95% CI: 0.547–0.641), and 6‐, 9‐, and 12‐month AUCs of 0.615, 0.624, and 0.633, respectively. The total Meet‐URO score and the three‐category IMDC classification yielded C‐indices of 0.607 and 0.538, respectively. The apparent COMP‐IS‐minus‐Meet‐URO risk‐group C‐index difference was 0.174 (95% CI, 0.124–0.224) in the paired analysis of fixed scores; this comparison was not optimism‐corrected (Table 4).

**TABLE 4:** Post hoc comparison with the published Meet‐URO score.

Panel A. Distribution of the original five Meet‐URO risk groups			
Risk group	Total score	Patients, *n* (%)	PFS events, *n*
1	0–1	39 (16.5)	13
2	2–3	103 (43.5)	52
3	4–5	72 (30.4)	40
4	6–8	22 (9.3)	18
5	9	1 (0.4)	1

Panel B. Discrimination of published score representations				
Score representation	C‐index (95% CI)	6‐month AUC (95% CI)	9‐month AUC (95% CI)	12‐month AUC (95% CI)
Meet‐URO: five risk groups	0.593 (0.547–0.641)	0.615 (0.535–0.696)	0.624 (0.553–0.692)	0.633 (0.567–0.703)
Meet‐URO: total score (0–9)	0.607 (0.558–0.655)	0.630 (0.556–0.714)	0.636 (0.562–0.707)	0.648 (0.579–0.720)
IMDC: three risk categories	0.538 (0.501–0.576)	0.537 (0.478–0.593)	0.532 (0.472–0.585)	0.550 (0.496–0.604)

Panel C. Paired differences in apparent C‐index	
Comparison	Difference in C‐index (95% CI)
COMP‐IS − Meet‐URO risk groups	0.174 (0.124–0.224)
COMP‐IS − Meet‐URO total score	0.160 (0.111–0.213)
Meet‐URO risk groups − IMDC score	0.028 (−0.012–0.072)
Meet‐URO risk groups − IMDC risk categories	0.055 (0.018–0.093)

*Note:* All analyses include 237 patients and 124 PFS events. Higher score values or risk categories indicate higher risk, whereas tied risks receive half of the credit. Meet‐URO weights and group boundaries are retained as previously described. The total Meet‐URO score and three‐category IMDC analyses are used for sensitivity analyses. Confidence intervals were obtained from 1000 paired patient‐level bootstrap resamples, with all scores held constant. Differences involving COMP‐IS refer to apparent discrimination and are conditional on the final fitted score; they do not include model selection uncertainty or constitute optimism‐corrected comparisons. Full‐pipeline‐corrected performance of COMP‐IS is reported in Panel C of Table 3. These comparisons are exploratory in nature.

Abbreviations: AUC, area under the curve; C‐index, concordance index; CI, confidence interval; COMP‐IS, composite inflammatory score; IMDC, International Metastatic Renal Cell Carcinoma Database Consortium; PFS, progression‐free survival.

### 3.4 Absolute‐Risk Performance and Exploratory Decision‐Curve Analysis

The linear predictor (LP) of the combined absolute‐risk model was LP = 1.247633749 × COMP‐IS + 0.183993889 × IMDC, with PFS(*t*|*x*) = *S*_0_(*t*)^exp(LP). Breslow baseline survival was *S*_0_ (6) = 0.855099990, *S*_0_ (9) = 0.775321485, and *S*_0_ (12) = 0.690891590. Apparent grouped calibration estimates tracked the identity line at the three horizons (Figure 3), and the internally corrected calibration slope was 0.928. Apparent IPCW Brier scores were 0.136, 0.144, and 0.152 at 6, 9, and 12 months, respectively. At 12 months, both COMP‐IS and IMDC + COMP‐IS had higher modeled net benefit than treat‐all and treat‐none strategies across the displayed threshold range of 0.10–0.60; the two model curves were closely aligned (Figure 4).

**FIGURE 3:**
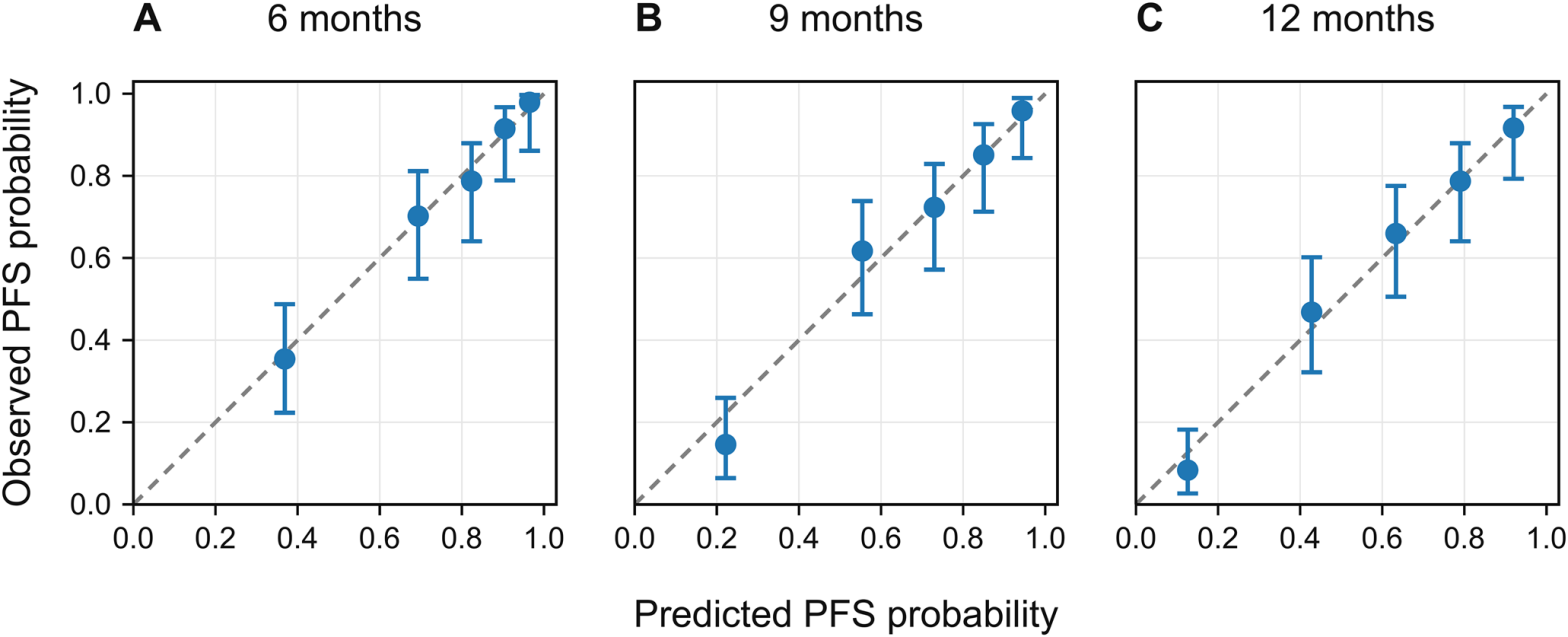
Apparent calibration of the IMDC+COMP‐IS absolute‐risk model at 6 months (A), 9 months (B), and 12 months (C). Points show quintile‐specific observed Kaplan–Meier PFS probabilities and vertical bars show pointwise 95% confidence intervals. The dashed line indicates ideal calibration. The internally corrected calibration slope is 0.928. COMP‐IS, composite inflammatory score; IMDC, International Metastatic Renal Cell Carcinoma Database Consortium; PFS, progression‐free survival.

**FIGURE 4:**
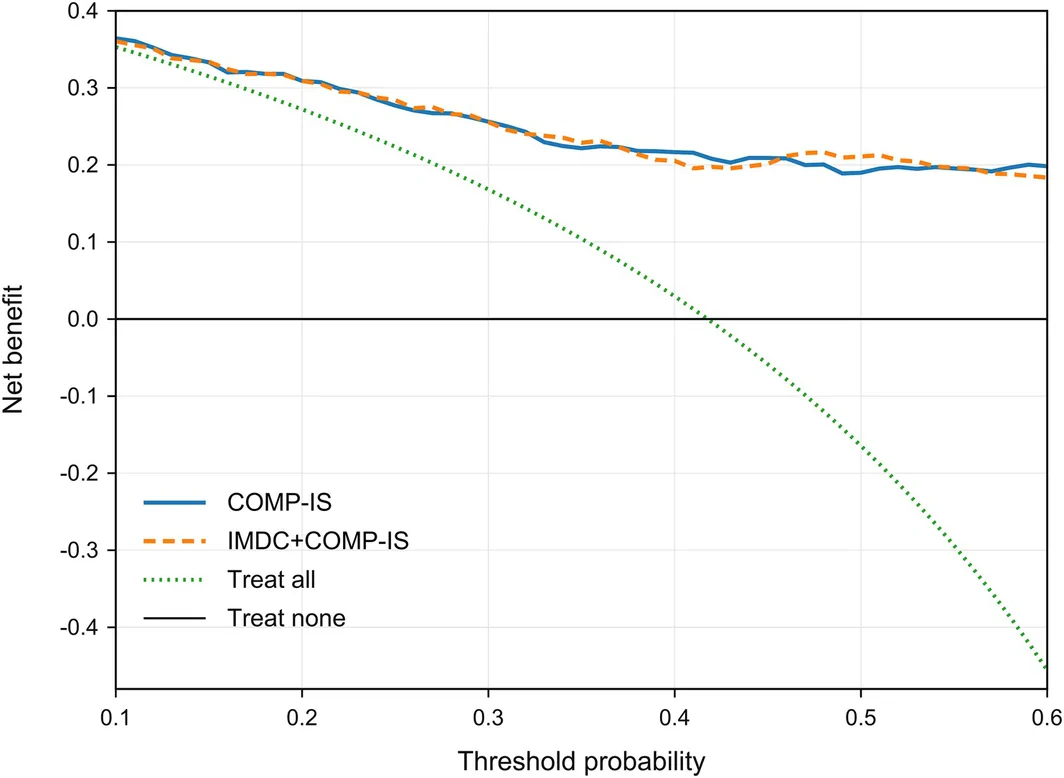
Exploratory decision‐curve analysis for progression or death by 12 months. Net benefit is shown across threshold probabilities of 0.10–0.60 for COMP‐IS, IMDC+COMP‐IS, treat‐all, and treat‐none strategies. Threshold probabilities refer to model‐predicted event risks, not the COMP‐IS ≥ 1.0 cutoff. Treat‐all and treat‐none represent hypothetical additional clinical actions, not administration or withholding of toripalimab plus axitinib. The curves do not establish the benefit of a specific intervention or validate a threshold for clinical implementation. COMP‐IS, composite inflammatory score; IMDC, International Metastatic Renal Cell Carcinoma Database Consortium.

### 3.5 Fixed‐Threshold Stratification and Secondary Outcomes

The COMP‐IS ≥ 1.0 rule classified 35 patients as high risk and 202 as low risk. Median PFS was 4.21 months in COMP‐IS‐High versus 22.42 months in COMP‐IS‐Low, with 12‐month PFS rates of 5.7% (95% CI, 1.0–16.7) and 67.3% (95% CI, 60.4–73.3), respectively (log‐rank *p* < 0.001; Table 3, Panel D; Figure 5).

**FIGURE 5:**
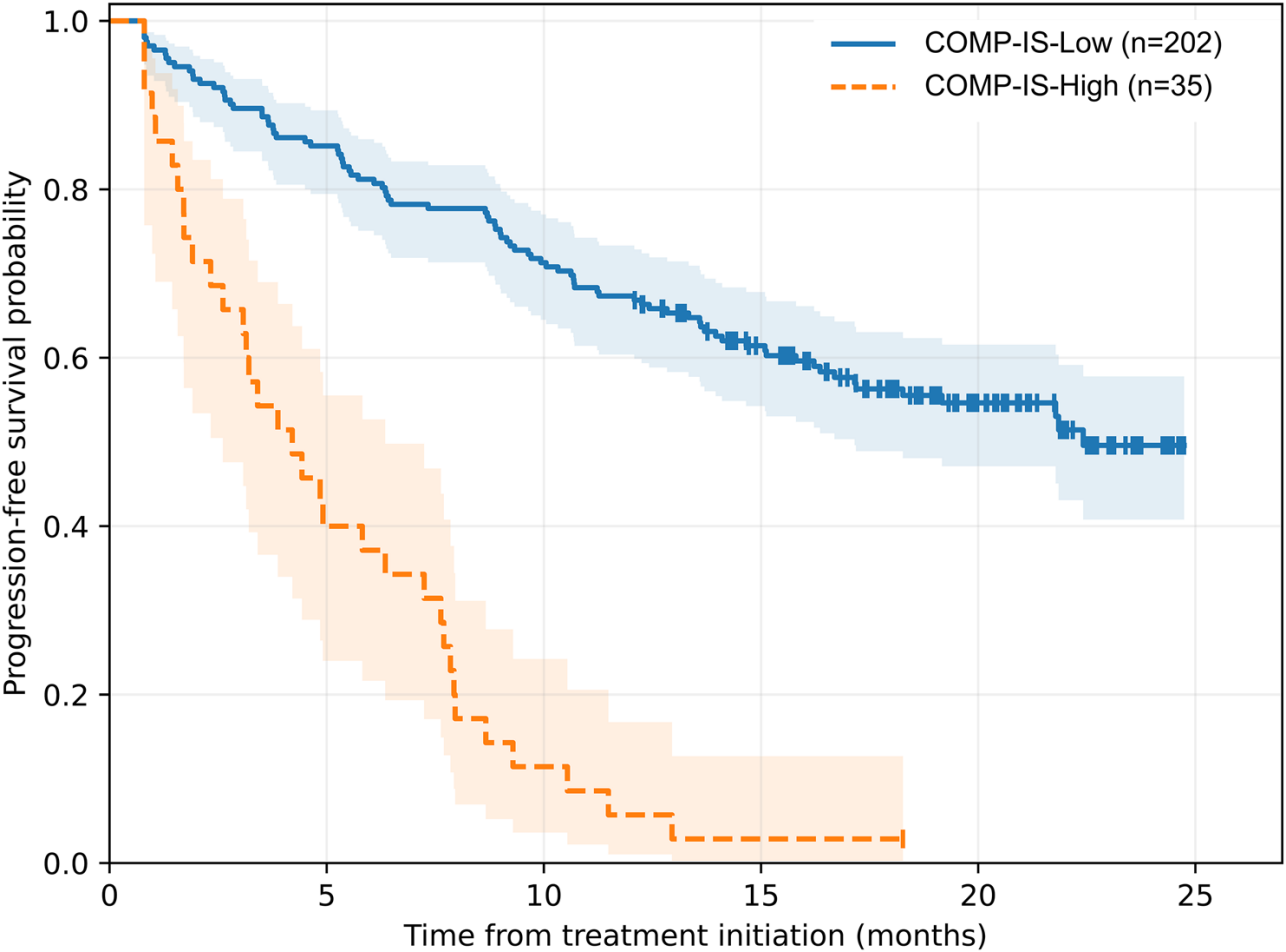
Kaplan–Meier estimates of progression‐free survival according to the fixed COMP‐IS threshold. COMP‐IS‐High was defined as COMP‐IS ≥ 1.0. Shaded areas indicate pointwise 95% confidence intervals. Vertical marks indicate censoring and match the corresponding curve. The threshold is not optimized against survival outcomes. COMP‐IS, composite inflammatory score.

ORR was 28.6% (10/35 patients) in COMP‐IS‐High and 57.4% (116/202 patients) in COMP‐IS‐Low (odds ratio, 0.30; 95% CI, 0.14–0.65; *p* = 0.002). DCR was 60.0% (21/35 patients) in COMP‐IS‐High and 88.1% (178/202 patients) in COMP‐IS‐Low (odds ratio, 0.20; 95% CI, 0.09–0.45; *p* < 0.001; Table 3, Panel E). Grade ≥ 3 adverse events and immune‐related adverse events did not differ significantly between COMP‐IS groups.

During a median follow‐up of 19.22 months for OS, 74 deaths were recorded. Kaplan–Meier‐estimated OS rates for the full cohort were 82.7% (95% CI, 77.3–87.0) at 12 months and 63.8% (95% CI, 56.3–70.4) at 24 months, with 196 and 17 patients remaining at risk, respectively. However, the 24‐month estimate should be interpreted with caution because of the small number of at‐risk patients. In the secondary OS model, each 1‐SD higher COMP‐IS was associated with shorter OS after IMDC adjustment (HR, 2.76; 95% CI, 2.17–3.51; *p* < 0.001).

Exploratory subgroup analyses showed no clear evidence of heterogeneity across the clinical categories examined (all interaction *p*‐values > 0.10; Figure 6).

**FIGURE 6:**
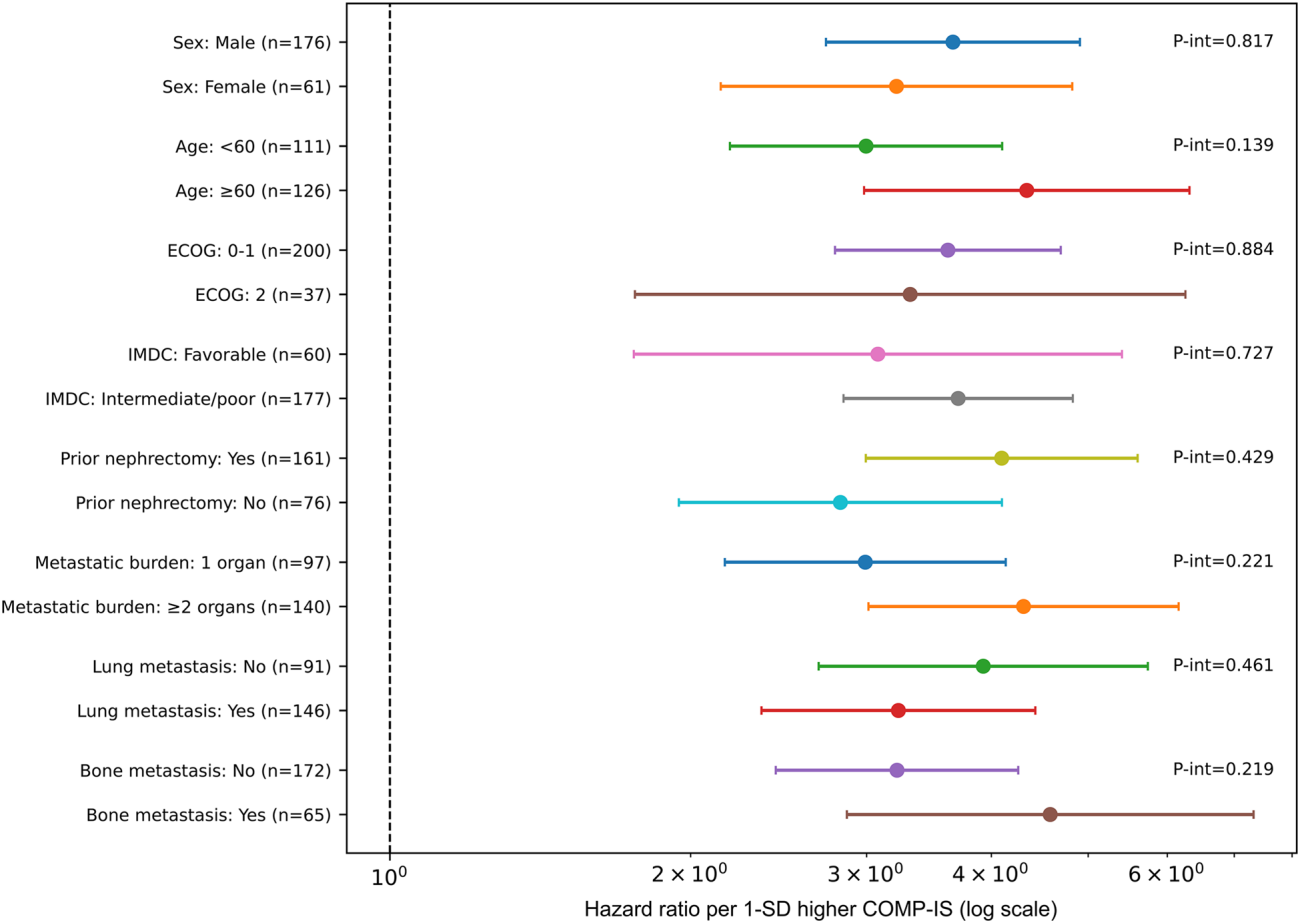
Exploratory subgroup associations of continuous COMP‐IS with progression‐free survival. Points and horizontal bars show hazard ratios and 95% confidence intervals per 1‐SD higher COMP‐IS. COMP‐IS‐by‐subgroup interaction *p*‐values are displayed at the right for each subgroup factor; no multiplicity adjustment was applied. COMP‐IS, composite inflammatory score; ECOG, Eastern Cooperative Oncology Group; IMDC, International Metastatic Renal Cell Carcinoma Database Consortium; SD, standard deviation. *p*‐int denotes the *p*‐value for interaction.

## 4 Discussion

In this study, we evaluated 10 overlapping inflammatory and nutritional indices within a common continuous‐variable framework and developed COMP‐IS using representatives from three domains. Discrimination was assessed using full‐pipeline bootstrap resampling with repeated transformations, marker selection, coefficient estimation, and score construction. Repeating these steps helps account for the optimism introduced by the data‐dependent model development. Within this cohort, COMP‐IS had a higher bootstrap‐corrected C‐index than the CAR or IMDC alone, whereas adding IMDC to COMP‐IS produced little additional discrimination. These findings support further evaluation of COMP‐IS alongside IMDC without establishing COMP‐IS as a replacement for the existing clinical framework.

This interpretation is relevant because the therapeutic landscape has advanced faster than the routine risk stratification. RENOTORCH established toripalimab plus axitinib as an effective first‐line option, whereas KEYNOTE‐426, CheckMate 9ER, and CLEAR demonstrated durable benefits from other ICI–TKI combinations [1, 2, 3, 4]. Despite these benefits, patients with apparently similar conventional risks may follow very different trajectories, and no routinely implemented biomarker currently guides selection among the major first‐line combinations [9]. Host response markers occupy the pragmatic space between conventional clinical scores and resource‐intensive molecular profiles. Blood counts, CRP, and albumin levels, and related laboratory measurements are embedded in standard care and can be repeated without additional tissue acquisition. Their clinical appeal lies less in mechanistic specificity than in the possibility that they summarize a dimension of tumor–host interaction that is incompletely represented by anatomical burden or categorical clinical risk.

The common‐framework comparison also helps reconcile the literature, in which individual inflammatory indices have often been studied separately. Contemporary RCC cohorts and meta‐analyses support prognostic associations for NLR, PLR, SII, and related inflammatory measures during immune‐based or systemic therapy [11, 12, 13, 14, 15]. In JAVELIN Renal 101, both NLR and CRP‐based analyses remained informative in an ICI–TKI setting [16, 17], while modified Glasgow prognostic scores and early CRP kinetics further support acute‐phase activity as a clinically relevant host signal [18, 19, 20]. The consistency of these associations across vascular endothelial growth factor–directed therapy, dual‐checkpoint blockade, and ICI–TKI combinations suggests that systemic inflammation may have prognostic relevance across treatment settings in advanced RCC. Furthermore, treatment context is important. Antiangiogenic therapy can alter vascular permeability, tissue hypoxia, and myeloid trafficking, whereas checkpoint inhibition changes immune activation and exhaustion states; therefore, the same circulating index may integrate different biological contributions at different treatment stages. This analysis refined the literature by reducing the differences caused by marker‐specific cut‐offs or transformations. The finding that COMP‐IS performed better than CAR suggests that CRP–albumin biology is important, but not exhaustive; leukocyte balance and nutritional reserve may contribute complementary information. Nevertheless, these results should not be interpreted as evidence that a more complex composite is invariably preferable; CAR remains a strong, simpler benchmark that should be retained in future external validation.

The biological coherence of COMP‐IS depends on the convergence of acute‐phase signaling, myeloid inflammation, lymphocyte availability, and systemic reserves. CRP is largely driven by interleukin‐6–signal transducer and activator of transcription 3 signaling, whereas albumin declines during sustained inflammatory and catabolic stress; therefore, CAR captures the contrast between acute‐phase activation and physiological reserve [32]. PNI integrates albumin with lymphocyte count and links nutritional reserve to immune cell availability, which is consistent with contemporary models that frame cancer cachexia as a systemic inflammatory and metabolic syndrome rather than a deficit of caloric intake alone [33]. Selection of the nutritional component was unstable across bootstrap resamples; PNI and ALI were selected in 47.9% and 52.1% of cases, respectively. PNI was retained in the full‐cohort model because its AIC was within two units of that of ALI, and it required fewer raw components. The near‐balanced selection frequencies indicated uncertainty in the exact composition of COMP‐IS and did not establish a consistently preferred nutritional marker. Repeating candidate selection during bootstrap estimates optimism associated with the development process but does not eliminate uncertainty about the performance of the final PNI‐based model in independent populations.

NLR contributes to a distinct axis centered on the myeloid–lymphoid balance. Neutrophils in cancer are heterogeneous and can promote immune suppression, extracellular trap formation, matrix remodeling, angiogenesis, and metastatic niche formation [34]. In clear cell RCC, single‐cell and spatial studies have documented progressive immune dysfunction and treatment‐associated reprogramming of the tumor and immune compartments [35, 36]. More recent work on programmed cell death protein 1 blockade combined with tyrosine kinase inhibition has identified remodeling of myeloid and lymphoid states [37], whereas spatial transcriptomic analyses have implicated secreted phosphoprotein 1–CD44 signaling in primary checkpoint inhibitor resistance [38]. Peripheral ratios cannot be equated with these intratumoral states, but a high NLR is biologically compatible with myeloid predominance and reduced lymphocyte reserve. Conversely, the clinical benefit of checkpoint blockade has been associated with the expansion of activated circulating CD8 T cells [39]. Therefore, a composite that integrates these systemic signals may capture a broader host phenotype than a single ratio.

A higher proportion of patients with an ECOG performance status 0 in the COMP‐IS‐High group should be interpreted with caution. Given the small size of this group and multiple descriptive baseline comparisons, this imbalance may reflect sampling variation. It should not be taken as evidence that a higher COMP‐IS score is biologically associated with better functional status.

The nonsignificant IMDC coefficient after adjustment for COMP‐IS should not be interpreted as evidence that IMDC lacks prognostic value or that it can be replaced. The small number of patients in the IMDC poor‐risk category (*n* = 3) also warrants caution when interpreting the results of this cohort. IMDC was retained in the combined model to maintain alignment with the established clinical prognostic framework, irrespective of its coefficient *p*‐value. Although adding IMDC to COMP‐IS produced little additional discrimination in this dataset, this comparison does not establish that COMP‐IS should supersede IMDC. Therefore, we consider COMP‐IS a candidate complementary prognostic measure to IMDC, and its added clinical value requires evaluation in independent populations.

Meet‐URO is a relevant comparator because it adds inflammatory and metastatic site information to IMDC and has been evaluated in first‐line immunotherapy combination cohorts [7, 8]. Its original development focused on overall survival in previously treated nivolumab recipients, whereas the present comparison assessed PFS during toripalimab plus axitinib treatment [6]. Although COMP‐IS showed higher apparent discrimination in this dataset, the paired comparison was conditional on its final fitted score and did not account for model selection uncertainty. Only one patient belonged to the highest Meet‐URO risk group, along with a limited representation of poor IMDC risk, constraining the assessment across the full prognostic spectrum. These findings support independent head‐to‐head evaluations and do not establish that COMP‐IS should replace IMDC or Meet‐URO.

The COMP‐IS‐High category comprised only 35 patients, which limited the precision of subgroup‐specific survival estimates, particularly later in follow‐up, when few patients remained at risk. Therefore, marked separation of the Kaplan–Meier curves should be interpreted cautiously. Although the COMP‐IS ≥ 1.0 threshold was not optimized for survival outcomes, it was applied to a score developed in the same cohort. This analysis should be regarded as descriptive risk stratification rather than an independent validation of a clinical decision threshold.

If externally validated, the COMP‐IS could be evaluated as an adjunct to the IMDC to identify patients who may warrant more frequent clinical reviews or earlier reassessment when progression is suspected. Other potential applications include structured nutritional and symptom assessments, referral for supportive care when indicated, and assessment of eligibility for appropriate clinical trials. However, these applications were not evaluated as COMP‐IS‐guided management strategies in this study. DCA estimated the net benefit under hypothetical decisions based on the predicted 12‐month event risk; it did not establish that any specific intervention improves outcomes or identify a validated threshold for implementing these actions. Therefore, prospective studies are needed to define the intended action, justify the corresponding risk threshold, and evaluate the clinical impact before the COMP‐IS is used to direct clinical care.

The strengths of the analysis include treatment homogeneity, uniform processing of 10 candidate indices, explicit mathematical specification of COMP‐IS and the absolute‐risk model, and internal validation that repeats the complete development pipeline. However, this study has limitations. The single‐center retrospective design made it susceptible to selection bias and residual confounding factors. Eligibility required complete pretreatment laboratory data and at least one posttreatment efficacy assessment; therefore, the analyzed cohort may not fully represent all patients starting this regimen. Differences in patient selection, laboratory measurements, and clinical practice across centers may also limit the transportability of the COMP‐IS. Bootstrap validation estimates optimism within the development data but cannot address these sources of selection bias or establish performance in other populations. Therefore, locked coefficients and standardization constants should be tested without re‐estimation in independent multicenter cohorts, considering laboratory platform differences and the recalibration of absolute risk. External studies should also compare COMP‐IS directly with CAR, IMDC, and Meet‐URO and assess the stability of nutritional domain selection between PNI and ALI. OS was a secondary endpoint, and the limited follow‐up, particularly the small number of patients remaining at risk at 24 months, warrants caution when interpreting the long‐term survival estimates. A longer follow‐up is needed to better characterize the association between COMP‐IS and OS. Serial measurements may determine whether changes in inflammatory–nutritional state add useful information beyond the baseline assessment.

In this single‐center cohort of patients with mccRCC treated with first‐line toripalimab plus axitinib, COMP‐IS had a higher bootstrap‐corrected C‐index for PFS than CAR or IMDC alone. The score should be interpreted as a prognostic model rather than a biomarker for treatment selection. However, COMP‐IS has only undergone internal validation, and its generalizability and clinical utility are yet to be established. Independent multicenter validation with recalibration, where necessary, and prospective evaluation of clinical usefulness are needed before routine clinical implementation.

## Funding

This work was supported by the Boxi Cultivation Plan of the First Affiliated Hospital of Soochow University (Grant number: BXQN2025004). The funder played no role in the study design, analysis, interpretation, or manuscript preparation.

## Ethics Statement

This study was approved by the Ethics Committee of the First Affiliated Hospital of Soochow University (No. 682(2026)). The requirement for written informed consent was waived because of the retrospective nature of the study and the use of de‐identified data.

## Conflicts of Interest

The authors declare no conflicts of interest.

## Data Availability

The data analyzed in this study are available at https://figshare.com/s/bd0ebe56469b9151cbd2.
